# Child Heritage Language Development: An Interplay Between Cross-Linguistic Influence and Language-External Factors

**DOI:** 10.3389/fpsyg.2021.651730

**Published:** 2021-11-11

**Authors:** Natalia Meir, Bibi Janssen

**Affiliations:** ^1^Department of English Literature and Linguistics, Bar-Ilan University, Ramat Gan, Israel; ^2^Independent Researcher, Amsterdam, Netherlands

**Keywords:** heritage language development, child bilingualism, cross-linguistic influence, input, case morphology, Russian, Hebrew, Dutch

## Abstract

The current study investigated the mechanisms of heritage language (HL) development with a focus on case morphology. First, the effects of cross-linguistic influence (i.e., the influence of the properties of the societal language (SL) on the acquisition of the HL) was assessed by performing bilingual vs. monolingual, and between-bilingual group comparisons (Russian–Dutch vs. Russian–Hebrew bilinguals). Russian, Hebrew, and Dutch show differences in the marking of the accusative (ACC) and genitive (GEN) cases, and these differences were used as a basis for the evaluation of cross-linguistic influences. Second, the study evaluated the contribution of language-external factors such as chronological age, age of onset of bilingualism (AoO), languages spoken by the parent to the child (only HL, only SL, both HL and SL), and family language type (both parents are HL speakers, mixed families). Finally, we assessed how language-external factors might potentially mitigate the effects of cross-linguistic influences in bilinguals. Russian-Dutch bilinguals from the Netherlands (*n* = 39, *M*_*AGE*_ = 5.1, *SD* = 0.8), Russian-Hebrew bilinguals from Israel (*n* = *36, M*_*AGE*_ = 4.9, *SD* = 0.9) and monolingual Russian-speaking children (*n* = *41, M*_*AGE*_ = 4.8, *SD* = 0.8), along with adult controls residing in the Russian Federation, participated in the study. The case production of ACC and GEN cases was evaluated using elicitation tasks. For the bilinguals, the background data on individual language-external factors were elicited from the participants. The results show that case morphology is challenging under HL acquisition—case acquisition in the HL is impeded under the influence of the properties of the SL. This is evident in the lower performance of both bilingual groups, compared with the monolingual controls who showed ceiling performance in the production of target inflection in the ACC and GEN contexts. More specifically, the acquisition of morphology is hindered when there are differences in the mapping of functional features (such as with Russian-Hebrew bilinguals) and/or the absence of this feature marking (such as with Russian-Dutch bilinguals). But the findings also point to the involvement of language-external factors as important mitigators of potential negative effects of cross-linguistic influence. In summary, HL development is an intricate interplay between cross-linguistic influence and language-external factors.

## Introduction

The term “heritage language” (HL)—also called “minority language,” “community language,” “home language,” “family language,” “mother tongue,” and “L1”—refers to a language that is spoken at home, but not by the majority of society which uses societal language (SL) (Rothman, 2009; Benmamoun et al., 2013; Montrul, 2016; Polinsky, 2018; Ortega, 2020). Numerous studies have investigated the end-state grammars of adult HL speakers (for an overview see Montrul, 2016; Polinsky, 2018; Polinsky and Scontras, 2019, 2020). The current study aims to contribute to the understanding of the mechanisms affecting child HL development. We used the term “child HL speakers,” but it should be noted that in previous research, the term “L1 development” in simultaneous or early sequential bilinguals has been used instead [for a detailed discussion on the terminology in HL and child bilingualism research see Kupisch and Rothman (Kupisch and Rothman, 2018)]. Today, more and more studies use the term “child HL speakers” (e.g., Meir and Armon-Lotem, 2015; Cuza and Pérez-Tattam, 2016; Daskalaki et al., 2019, 2020; Chondrogianni and Schwartz, 2020; Goebel-Mahrle and Shin, 2020; Rodina et al., 2020; Serratrice, 2020; Armon-Lotem et al., 2021; Otwinowska et al., 2021). Heritage language speakers acquire their HL from birth *via* naturalistic input, but as adults, they show divergence from the baseline (the language spoken in the country of origin, or the language spoken by the first generation of immigrants who are dominant in this language). Among the possible mechanisms triggering divergences in HL grammars, researchers have proposed cross-linguistic influences from the SL and/or language-external factors (such as the age of onset of bilingualism (AoO), quantity and quality of exposure to HL and SL, family type (both parents are HL speakers vs. a mixed HL-SL family), HL community size, and the number of HL speakers in the child‘s environment). For a detailed review of the potential mechanisms affecting HL development see Montrul (2016), Polinsky (2018), and Polinsky and Scontras (2019, 2020).

There is no agreement on the underlying mechanisms affecting morphosyntactic development in child HL speakers. Some studies reported that child HL speakers pair up with monolingual peers on morphosyntax. Alternatively, studies showed that child HL speakers diverge from their monolingual peers in this domain. Some researchers attribute these divergences to cross-linguistic influences (for a detailed overview on cross-linguistic influence in child bilingualism see Serratrice, 2013; Van Dijk et al., 2021), while some studies found that cross-linguistic influences did not affect the HL acquisition of morphosyntax, suggesting that language-external factors shape child HL development (e.g., Daskalaki et al., 2019, 2020; Rodina et al., 2020). Cross-linguistic influences and language-external factors might not be mutually exclusive and shape HL development together (Daskalaki et al., 2019, 2020; Van Dijk et al., 2021). In the current study, we evaluate the involvement of both factors.

To contribute to this ongoing debate, two groups of bilinguals, who acquired Russian as their HL in contact with two typologically different SLs (Dutch and Hebrew), were compared with their monolingual Russian-speaking peers regarding the production of case inflectional morphology. We compared the bilinguals with their monolingual peers, and we compared the two bilingual groups which acquired the same HL with typologically different SLs. The choice of case inflectional morphology was motivated by the fact that Russian is a language with a rich case morphology that realizes case features morphologically. Dutch does not mark cases on nouns, while Hebrew marks the accusative (ACC) cases with the dedicated particle “*et*” in front of definite noun phrases. Neither Dutch nor Hebrew uses special morphology to mark genitive (GEN) cases in negative constructions. These properties of SL-Dutch and SL-Hebrew might potentially impede the acquisition and maintenance of case morphology in HL-Russian of bilingual children. Thus, the comparison of two different bilingual groups, whose SLs differ in marking cases, is expected to shed light on whether SL properties affect the HL development of a child, and how language-external factors shape HL development and mitigate the potential negative effects of the SL on the HL.

In this introductory section, we first review studies assessing cross-linguistic influence in HL development. Second, we discuss the effects of language-external factors on HL development. Third, we provide a brief overview of the case morphology in the Russian Language (the HL of the bilingual children in the current study), and then we discuss the typological differences between Russian, Dutch, and Hebrew focusing on case marking in the three languages. We conclude the introductory section with the research questions and hypotheses of the current study.

### Factors Affecting Child Heritage Language (HL) Acquisition

#### Cross-Linguistic Influence in Child HL Acquisition

Cross-linguistic influence has been proposed to account for the success or failure of bilinguals in their acquisition of certain linguistic properties in one language in the presence of the other language. A facilitative effect is observed when the properties of the two languages have converging configurations. A negative cross-linguistic influence (also called cross-linguistic transfer/interference), defined as “deviation from the norms of either language which occur in the speech of bilinguals as a result of their familiarity with more than one language” (Weinreich, 1968), has been robustly demonstrated in the acquisition of L2/SL in simultaneous and sequential bilingual children (e.g., Zdorenko and Paradis, 2008; Blom et al., 2012). A recent meta-analysis which evaluated cross-linguistic influence and its predictors in 750 simultaneous and early sequential bilingual children (aged 4;0–10;0) in 17 unique language combinations across 26 experimental studies confirmed the presence of cross-linguistic influences in bilingual morphosyntactic acquisition (see Van Dijk et al., 2021).

Several hypotheses have been suggested to account for the cross-linguistic influences in various bilingual child and adult populations (Blom et al., 2017). Some propose that cross-linguistic influence occurs when there is a partial structural overlap between the two languages, while cross-linguistic influence is not predicted when the language structures are either completely distinct or completely overlap (Hulk and Müller, 2000). The *feature (re)-assembly hypothesis* (Lardiere, 2009) proposes that not only the absence/presence of a feature in the two languages determines learnability but also the differences in the mapping and bundling of features in the L1 and the L2 shape the trajectory of acquisition. A facilitative effect is expected if the properties are similarly mapped in the L1 and L2, while an impeding effect is expected when the properties in the two languages have different configurations. The predictions of some cross-linguistic influence hypotheses, originally proposed for L2 acquisition, have been extended to adult and child HL acquisition as well. For example, the *feature (re)-assembly hypothesis* (Lardiere, 2009) has been extended to child HL acquisition (see Cuza and Pérez-Tattam, 2016; Meir et al., 2017), showing that feature (re)-assembly in the HL is affected by the properties of the SL.

Individual studies evaluating the effects of cross-linguistic influence on child HL acquisition provide inconclusive evidence. There is accumulating evidence that SL properties affect HL development (e.g., Guiberson et al., 2006; Gathercole and Thomas, 2009; Meir et al., 2017). Most of the previous studies evaluating cross-linguistic influence in HL development have employed a monolingual vs. bilingual comparison paradigm. For example, Montrul and Sánchez-Walker (2013) showed evidence for the influence of SL-English on HL-Spanish in child heritage speakers aged 6;0–17;0 for the Spanish differential object marker *a*. Child heritage speakers of Spanish were reported to omit the differential object marker significantly more frequently than their monolingual peers. This has been linked to the influence of SL-English which does not have a differential object marker. Similarly, Cuza and Pérez-Tattam (2016) compared two groups of Spanish-speaking children (aged 5;0–10;8): HL-Spanish and SL-English speakers, and monolingual controls. The authors reported significant differences between the two groups. Furthermore, the authors attributed the decreased accuracy on gender agreement and gender assignment in HL-Spanish to the properties of SL-English which do not mark grammatical gender. In the same vein, Meir et al. (2017) compared HL-Russian speakers with their monolingual controls and reported an asymmetry in the performance on ACC case accuracy and subject-verb agreement accuracy. The HL speakers paired up with their monolingual peers on subject-verb agreement, but they were less accurate in terms of the ACC case morphology. This asymmetry in their performance has been linked to the properties of SL-Hebrew; Hebrew marks gender, number, and person features on verbal inflections similarly to Russian, while the marking of case morphology is different in the two languages. Again, the lower accuracy on the ACC case of HL-Russian speakers was linked to the SL properties, suggesting cross-linguistic influence. It is also important to note that bilingual children might show an asymmetry between the comprehension and production of the same grammatical phenomenon. For example, Kim et al. (2018) showed that HL-Korean bilingual children aged 8;0–10;0 were less accurate in their comprehension of object-subject-verb (OSV) sentences as compared with their monolingual peers. The gap in comprehension might be attributed to the lack of ACC marking in English and the word order properties of English. But on the production tasks, the same children correctly produced the ACC marking. A meta-analysis by Van Dijk et al. (2021) compared the magnitude of cross-linguistic influence in 26 studies between bilinguals and their monolingual peers on a wide range of morphosyntactic structures and concluded that “cross-linguistic influence is part and parcel of being bilingual and can manifest itself in various linguistic contexts.” The authors furthermore concluded that the magnitude of cross-linguistic influence is stronger from the SL to the HL, which is crucial for the predictions of the current study, as our study focuses on the influence of two typologically different SLs on HL-Russian.

Another paradigm that is used to evaluate cross-linguistic influence is the comparison of several bilingual groups, rather than the bilingual vs. monolingual comparison (e.g., Sorace et al., 2009; Schwartz et al., 2015; Janssen and Meir, 2019; Kaltsa et al., 2020; Rodina et al., 2020). Such studies were not included in the meta-analysis by Van Dijk et al. (2021) due to their scarcity. Studies employing multiple bilingual group comparisons bring inconclusive results. On the one hand, there are studies confirming the influence of SL on HL. For example, Schwartz et al. (2015) compared the noun–adjective gender agreement in HL-Russian with various SLs (English, Finnish, German, and Hebrew). The authors reported the cross-linguistic influence of the SL properties on HL gender acquisition/maintenance. The HL-Russian speakers whose SL marks grammatical gender (German, Hebrew) outperformed the bilinguals whose SL does not mark grammatical gender (English, Finnish) on gender agreement in HL-Russian. On the other hand, a study by Rodina et al. (2020) also compared the noun–adjective gender agreement in HL-Russian speakers with varying SLs (German, Hebrew, Norwegian, Latvian, and English) and found that cross-linguistic influence did not affect HL acquisition. Similar to the study by Schwartz et al. (2015), the study by Rodina et al. (2020) included languages that had similar configurations to Russian concerning gender assignment/marking and languages which did not mark grammatical gender. Russian has a three-way gender system, as do German and Norwegian; Hebrew and Latvian have two-gender systems, while English does not mark grammatical gender at all. Yet the study by Rodina et al. (2020) found no evidence for the influence of SL on HL development. Rather, language-external factors were reported to shape HL grammatical gender acquisition.

To sum up, based on monolingual vs. bilingual comparisons, the presence of cross-linguistic influence has been shown for both SL and HL acquisition (see Van Dijk et al., 2021). More specifically, the lower accuracy on morphosyntax in the HL may be traced back to the influence of the SL, and the magnitude of cross-linguistic influence is higher from the SL to the HL, as compared with the cross-linguistic influence in the opposite direction. However, some studies show no effect of cross-linguistic influence, especially based on between-bilingual-group comparisons, but such studies are less frequent. The latter studies show that language-external variables rather than cross-linguistic influence shape HL development. In the next subsection, we focus on the role of language-external variables in HL development.

#### Language-External Factors in Child HL Acquisition

Language-external factors shape monolingual as well as bilingual language acquisition in both languages (see Unsworth, 2013, 2014, 2016, 2019; De Houwer et al., 2018; Armon-Lotem and Meir, 2019). The effects of various language-external factors have been evaluated in HL development: chronological age, longer period of uninterrupted HL acquisition (as indexed by AoO), greater use of HL at home, the size of the HL community, and current and cumulative exposure to HL predict the success of HL development (e.g., Daskalaki et al., 2019, 2020; Rodina et al., 2020; Armon-Lotem et al., 2021; Otwinowska et al., 2021), yet the specific details of the findings vary.

We begin our discussion with chronological age. Although the effect of chronological age is robust in monolingual typical language development, it is not always observed in bilingual HL development (Lein et al., 2017; Gagarina and Klassert, 2018). On one hand, studies have shown that HL skills improve with age, but on the other hand, it has also been demonstrated that HL competence shows fossilization and even declines with age. For example, Gagarina and Klassert (2018) showed the effects of age for the acquisition of HL-Russian morphosyntax in Russian-German bilinguals. Additionally, Cuza et al. (2021) showed that while child HL-Spanish speakers diverge on copular production, this divergence is diminished in adult HL speakers. However, a meta-analysis investigating the effects of cross-linguistic influence in child morphosyntactic acquisition found that this influence did not significantly change over time (see Van Dijk et al., 2021), suggesting that the effect of cross-linguistic influence is present in older bilingual children as well.

Furthermore, chronological age in bilinguals might be related to input/exposure characteristics and language dominance characteristics: some HL speakers continue to receive input in their HL, some HL speakers receive more input/exposure in the SL. Indeed, HL input/exposure quantity is an important predictor of the success/failure of HL acquisition (for an overview see Armon-Lotem and Meir, 2019). The input/exposure of HL diminishes as HL speakers get older and socialize using the SL (e.g., Unsworth, 2014; De Houwer et al., 2018), which might explain the fossilization and attrition/loss over time. For example, the HL input quantity at home is related to HL development: child HL-Greek speakers with <25% of HL use had very low accuracy rates on the morphosyntactic measures (Daskalaki et al., 2019). The AoO of SL is linked to HL morphosyntactic development: children with later SL AoOs, i.e., children with longer periods of uninterrupted HL acquisition, are shown to perform better on the morphosyntax compared with HL child speakers with earlier AoOs of SL (Meir et al., 2017; Armon-Lotem et al., 2021). Rodina et al. (2020) showed that several language-external factors such as family type (mixed family vs. HL-speaking parents), age, and current exposure to HL instruction are related to the HL development of grammatical gender in HL speakers with various SL properties. In contrast, Tsinivits and Unsworth (2021) showed that having an older sibling is related to the increased input of SL in the home setting, and hence higher SL abilities, yet having an older sibling was reported to have no effect on HL grammatical complexity. It should be noted that the input/exposure factors are related to language dominance/proficiency—children with more exposure/input to the language show higher dominance in that language (e.g., Treffers-Daller, 2019).

In addition to the diminished input quantity that HL speakers receive compared with their monolingual peers, the input quality is shown to be different from what monolinguals receive in the country where their language is dominant (e.g., Rothman, 2007; Montrul and Sánchez-Walker, 2013). HL speakers are not schooled in their HL; thus, their HL input is limited to home contexts (Montrul, 2016; Polinsky, 2018). For example, Daskalaki et al. (2020) showed that child HL-Greek speakers indeed diverge from monolingual Greek-speaking children on subject placement. The divergence from the monolingual controls was attributed not only to the decreased input that the HL-Greek speakers were receiving compared with monolingual controls but also to the divergences which were already visible in the speech of the parents of these children, showing that HL speakers are exposed to divergent inputs.

Thus, numerous language-external factors have been proposed to account for HL development in bilingual children. In the current study, we tested the contribution of cross-linguistic influences and language-external factors to shed light on the mechanism of HL development. Cross-linguistic influences and language-external factors might not be mutually exclusive (Daskalaki et al., 2020), and all contribute to HL development. Indeed, some scholars advocate that “input (quality/quantity) is not the only factor determining HL acquisition” (see Putnam and Sánchez, 2013, p. 487). Therefore, in the current study, we will consider both factors, language-external factors which index the quantity and quality of input, as well as cross-linguistic influences. Putnam and Sánchez (2013) proposed that the (re)-assembly of features in the HL (Lardiere, 2009) is related to the activation of a specific HL feature in production and comprehension. The HL feature activation might be related to cross-linguistic and language-external competitions which might result in its insufficient activation and gradual replacement by SL features. Before presenting the research questions of the study, we will briefly review the case morphology in Russian, Dutch, and Hebrew, which is the phenomenon that serves as a test case for the comparison between the monolingual and HL morphosyntactic development.

### Case Morphology in Russian, Dutch, and Hebrew

Russian is a language with a rich nominal inflectional morphology. Russian realizes case features morphologically: all Russian nouns, adjectives, quantifiers, demonstratives, and numerals must bear a dedicated case inflection (e.g., Bailyn, 2012). There are six main cases in Russian in singular and plural: nominative (NOM), GEN, ACC, dative (DAT), instrumental (INSTR), and prepositional (PREP). In the current study, we will focus on two cases: the ACC case of direct objects and the GEN case in negative constructions.

The choice of ACC and GEN case forms for the current study was not accidental. While Russian has a rich nominal morphology, Dutch and Hebrew have no case inflections. In Hebrew, the use of the ACC marking is limited (only in front of definite nouns), while in Dutch there are no special markings in the investigated constructions. These properties of the three languages will serve as a testing point to shed light on the mechanisms of HL development.

Table 1 lists the ACC and GEN case paradigms for singular nouns across the first and second declension classes for animate and inanimate nouns. In some nouns, the ACC and GEN case marking is visible, i.e., ACC/GEN are non-homophonous to NOM ones. For example, the noun *devočka* “girl” has three different forms for the NOM—ACC—GEN (e.g., *devočk**-a**- devočk**-u -*** *devočk**-i***). However, Russian is characterized by extensive morphological case syncretism. For example, for some nouns, ACC and GEN forms are homophonous to their NOM forms: the noun *mjaso* “meat” has three forms that overlap in their phonological form. For the word *stol* “table,” the ACC and the NOM forms overlap, while the GEN form is different (e.g., *stol – stol – stol-a*).

**Table 1:** The Russian case inflections ([NOM] → [ACC] → [GEN]) across 2 declension classes for animate and inanimate nouns.

		**English Translation**	**NOM**	**ACC**	**GEN**
1st Declension	FEM +Animate	*dog*	*sobak-**a***	*sobak-**u***	*sobak-**i***
	FEM -Animate	*star*	*zvezd-**a***	*zvezd-**u***	*zvezd-**y***
	MASC +Animate	*father*	*pap-**a***	*pap-**u***	*pap-**y***
2nd Declension	MASC -Animate	*table*	*stol*	*stol*	*stol-**a***
	NEUT -Animate	*window*	*okn-**o***	*okn-**o***	*okn-**a***
	NEUT -Animate	*butter*	*masl-**∂***	*masl-**∂***	*masl-**∂***

Nominative case forms are the most frequent in the Russian National Corpus (30.5%), followed by ACC forms, which comprise 20% of the total nominal inflections and 23% for GEN forms for singular nouns (as per the frequency data provided by Slioussar and Samoilova, 2015). Thus, it is plausible to suggest that form frequency should not trigger differences in the acquisition trajectory in monolingual and bilingual children. To the best of our knowledge, there is no available frequency analysis for the case form distributions in monolingual and bilingual Russian child-directed speech. Yet, looking into the data on Polish, a typologically close language to Russian, in early childhood (around age 2), about 54% of the nouns in child-directed speech are in the NOM form, 19% in the ACC, and 12% in the GEN (Dabrowska and Szczerbiński, 2006). It is plausible to suggest that these percentages observed for Polish might be similar in Russian as well, yet future studies should investigate the distribution of different case forms in Russian monolingual and bilingual child-directed speech.

Having presented the Russian case system, we now turn to Dutch and Hebrew, both of which have sparse case morphology, but the two languages show fundamental differences with respect to case marking. Dutch does not mark cases on nouns, whereas Hebrew marks the ACC case by the particle *et* only before definite nouns (Berman, 1978) and uses possessive GEN inflectional markers. In negative constructions, there is no special marker on the noun in Hebrew. Below, we present a comparison between Russian, Dutch, and Hebrew for the ACC (see 1) and GEN (see 2) environments investigated in the current study.


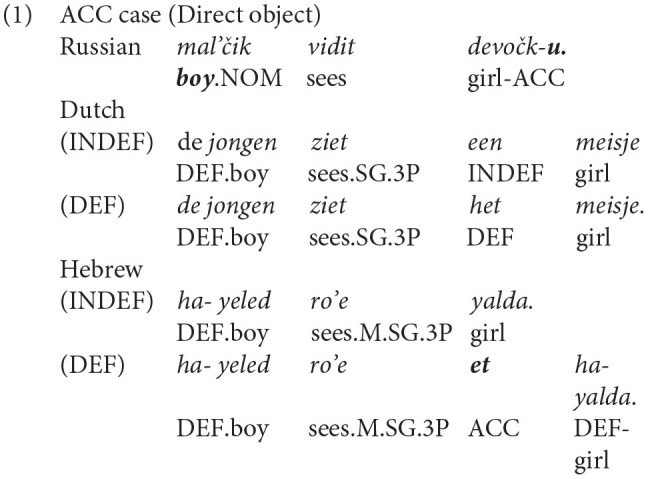



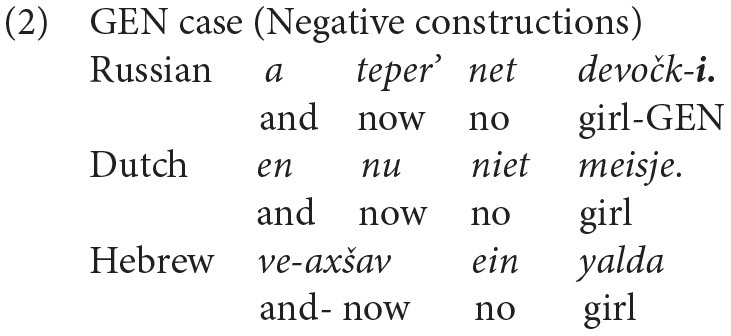


## Russian Case Acquisition Among Monolingual and Bilingual Children

Monolingual Russian-speaking children acquire the basics of the case system within a very short period (e.g., Gvozdev, 1961; Babyonyshev, 1993; Protassova and Voeikova, 2007; Cejtlin, 2009). Based on previous case studies, it is known that the NOM—ACC oppositions occur at about 1;9 (Gvozdev, 1961). Babyonyshev (Babyonyshev, 1993) showed that monolingual Russian-speaking children have full mastery of NOM (597 out of 600) and ACC (27 out of 30) cases from the moment of appearance of the arguments that require them. Furthermore, Babyonyshev (1993) found no errors with the GEN of negation in the speech of monolingual Russian-speaking children. Babyonyshev (1993) argued that the use of NOM, ACC, and GEN (in negation constructions) is already operative in monolingual Russian-speaking 2-year-olds.

Unlike monolingual children who show almost error-free production of case morphology from early on, child HL-Russian speakers are reported to have difficulties with case morphology. In particular, this seems to be the case when their SL has sparse case morphology (like in English, Dutch, Swedish) and/or realizes cases differently, e.g., using particles (like in Hebrew) or marking case on determiners (like in German) (see Turian and Altenberg, 1991; Gagarina, 2011; Ringblom, 2012; Schwartz and Minkov, 2014; Janssen, 2016; Meir et al., 2017; Gagarina and Klassert, 2018; Janssen and Meir, 2019; Armon-Lotem et al., 2021). It should be noted that the studies differ with respect to the elicitation methods tapping into case morphology (e.g., *spontaneous speech*: Gagarina, 2011; Ringblom, 2012; Schwartz and Minkov, 2014; *experimental elicitation tasks*: and Janssen, 2016; Janssen and Meir, 2019; Armon-Lotem et al., 2021, *sentence repetition tasks*: Meir et al., 2017), and with respect to the number of bilingual participants, ranging from case studies (Gagarina, 2011; Ringblom, 2012) to group comparisons (Janssen, 2016; Meir et al., 2017; Janssen and Meir, 2019; Armon-Lotem et al., 2021).

There is also evidence that the HL-Russian case system can be developed in a monolingual-like manner. For example, a study, investigating language development in a simultaneous bilingual child acquiring HL-Russian and SL-Turkish between 2;11 and 4;0, showed that the acquisition of case morphology is similar to that of monolingual children despite the reduced input of HL-Russian (Antonova Ünl and Li, 2018). It should be kept in mind that Turkish, the SL of the child, is an agglutinating language with rich case morphology. Turkish marks NOM, ACC case with definite noun phrases, DAT, LOC, GEN, and ablative case using dedicated case inflections. In the same vein, based on the data from an experimental task from 10 Russian-Finnish bilinguals aged 4;0–5;0, quantitative differences in HL-Russian in contact with Finnish were reported, but no qualitative differences were observed (Protassova et al., 2017). Russian-Finnish bilinguals rarely substituted various inflected case forms with the NOM default form.

To sum it up, while monolingual Russian-speaking children show almost error-free case production from early on, there is a conflict in the results for child HL-Russian speakers. On the one hand, some studies show monolingual-like error-free production in bilingual HL-Russian-speaking children. On the other hand, some studies point to profound difficulties with case morphology in bilingual HL-Russian-speaking children. There are several open questions that our study aims to answer. To what extent is case acquisition in the HL impeded/facilitated due to cross-linguistic influence from the SL, as has been proposed by previous studies? And to what extent do language-external factors mitigate possible negative cross-linguistic effects?

## Present Study

The current study aimed to investigate bilingual HL development by evaluating the mechanisms which might shape morphosyntactic HL development. We considered the effects of cross-linguistic influence, i.e., influence from the SL, and the effect of language-external factors, such as age, AoO of SL, home language use, family type, HL and SL input quantity, and HL and SL proficiency. Furthermore, we aimed to evaluate the interaction between these two potential mechanisms. Our specific research questions were as follows:

### RQ1: Is There an Effect of Cross-Linguistic Influence on Bilingual HL Development?

To address RQ1, we first employed bilingual vs. monolingual comparisons. Second, we compared the two bilingual groups between themselves (Russian-Dutch and Russian-Hebrew) to shed light on the cross-linguistic influence in HL acquisition i.e., the effect of the SL properties on the HL. Under the null hypothesis, we expected child HL speakers to perform on a par with their monolingual peers. The HL speakers were exposed to their HL from birth and acquired their HL *via* naturalistic input, thus, all groups might perform the same. We expected no group differences for the ACC and GEN forms which are homophonous to NOM, since, in both SLs (Dutch and Hebrew), the noun forms across different syntactic environments remain homophonous to the NOM form. Furthermore, under this hypothesis, no monolingual-bilingual differences in error patterns were expected.

Under the alternative hypothesis, we expected group differences. More specifically, group differences were predicted for the ACC and GEN forms which are non-homophonous to NOM, i.e., ACC and GEN forms that require the use of a dedicated infection. Group differences and error patterns were expected to shed light on the mechanisms of HL development. If the presence and absence of the features in one language regulates the patterns of acquisition in the other language, Russian-Hebrew bilinguals were predicted to show better mastery of the ACC in HL-Russian as compared with the GEN case, as Hebrew marks the ACC case. Thus, under this scenario, the presence of ACC in Hebrew should facilitate the acquisition of ACC in HL-Russian. In the case of Russian-Dutch bilinguals, such asymmetry was not expected, as Dutch does not have any morphological markings for any of these environments. Alternatively, based on the *feature (re)-assembly hypothesis* (Lardiere, 2009), we would expect no asymmetry between ACC and GEN in the bilingual groups, since the ACC case is mapped onto different lexical categories in the two languages of Russian-Hebrew bilinguals (onto case inflection in Russian and particle *et* in Hebrew), thus, these differences in realization are expected to impede the acquisition/maintenance of ACC in Russian-Hebrew bilinguals. Similarly, no asymmetry was expected in HL-Russian among Dutch bilinguals. Finally, if cross-linguistic influence shapes HL acquisition, we expected qualitative differences between monolinguals and bilinguals.

### RQ2: Do Language-External Factors Affect Bilingual HL Development?

To address RQ2, we aimed to evaluate whether age, AoO of SL, home language use, family type, HL and SL input quantity, and HL and SL proficiency affects the acquisition of case morphology in bilingual HL-Russian development. Previous research brings robust evidence for the key role of language-external factors for the acquisition of vocabulary in HL and SL, yet, with respect to the acquisition of HL morphosyntactic properties, the findings are less robust. Some studies show that various language-external factors might shape morphosyntactic HL development, while other studies fail to detect the effect of language-external factors (e.g., Daskalaki et al., 2019, 2020; Rodina et al., 2020; Armon-Lotem et al., 2021; Otwinowska et al., 2021).

In the current study, we did not consider qualitative input factors, although previous studies reported parental HL input divergences from the one provided by monolingual baseline speakers (e.g., Daskalaki et al., 2020). Immigrants constantly mix language in the same utterance, including Russian-speaking immigrants (e.g., Remennick, 2003). However, the Russian-speaking immigrants of the first generation inflect for case code-switched lexical items inserted into a Russian sentence, following the Russian system of case assignment based on declension classes. The correct assignment of cases (even on code-switched words) is indicative of intact grammatical structure in Russian. Furthermore, two recent studies showed no traces of divergences in case production and comprehension in Russian among first-generation immigrants (see Meir and Polinsky, 2021). Thus, based on previous studies, input type divergences were not expected to account for the ACC and GEN case differences in child HL.

### RQ3: Do Language-External Factors Mitigate the Potential Effects of Cross-Linguistic Influence?

To contribute to our understanding of the interplay between cross-linguistic influence and language-external factors, we assessed whether language-external factors mitigate the effects of cross-linguistic influence. If language-external factors mitigate cross-linguistic influence effects, we expected interactions between language-external factors and group membership (biDU or biHE). The interactions between language-external factors and groups membership were expected to show the differential effects of SL properties across the two cases, ACC and GEN, given the inherent differences of Dutch and Hebrew. The presence of an interaction between language-external factors and cross-linguistic inputs would support the claim that input is not the only factor shaping HL development. The interaction between the language-external and cross-linguistic influence would point to an interplay between these two factors suggesting that HL development is a complex process influenced by multiple mechanisms.

## Methodology

### Participants

Three groups of children and a group of monolingual adult controls participated in the study. The adult monolingual Russian-speaking controls (*n* = 10) were recruited from the Russian Federation. The adult controls were between the ages of 18 and 45, residing in Saint Petersburg. The monolingual child controls (monoRU) were recruited from the Russian Federation, while the bilingual HL-Russian speakers were residing in the Netherlands (biDU) or Israel (biHE). The background information on the child data is presented in Table 2. The current sample (*n* = 115) partially overlaps with the sample of children (*n* = 72) reported by Janssen and Meir (2019) that compared the accuracy production, comprehension, and repetition of the ACC case forms. In the current study, we investigated two cases: ACC and GEN.

**Table 2:** Background information on the participants per group.

		**biDU (*n* = 39)**	**biHE (*n* = 35)**	**monoRU (*n* = 41)**
Age (months)	*M (SD)* *MIN-MAX*	61 (8) 48–77	57 (9) 43–70	56 (8) 41–71
Length of uninterrupted HL acquisition (as measured by AoO)	*M (SD)* *MIN-MAX*	7 (11) 0–39	27 (20) 0–48	n/a
Length of exposure (months) to the Societal Language (SL)	*M (SD)* *MIN-MAX*	54 (13) 26–77	30 (18) 0–69	n/a
Mother's Education (% of mothers with a university degree)	%	95%	63%	83%
Language spoken at home		Only HL-Russian: 33.3% HL and SL: 66.7%	Only HL-Russian: 54.3% HL and SL: 45.7%	Only Russian: 100%
Parental ratings of the HL-Russian skills	*M (SD)* *MIN-MAX*	2.78 (0.82) 1–4	2.81 (0.70) 1–4	n/a
Parental ratings of the SL skills	*M (SD) MIN-MAX*	3.35 (0.77) 1–4	2.46 (1.07) 1–4	n/a
Current exposure to the HL-Russian (0–1)	*M (SD)* *MIN-MAX*	0.35 (0.18) (0–0.75)	0.58 (0.24) (0.25–1.00)	n/a
Family type	%	HL: 35% mixed: 65%	HL: 77% Mixed: 23%	n/a

There were significant group differences for age [*F*_(2, 112)_ = 3.67, *p* = 0.030, η^2^ = 0.062]: the biDU group was significantly older than the monoRU group (*p* = 0.031) as determined by the Bonferroni *post-hoc* analysis for pair-wise comparisons; no differences for age were detected between the biHE and the monoRU (*p* = 1.00), and between the two bilingual groups (*p* = 0.17). Similarly, there were significant differences between the groups with respect to the level of education of their mothers [χ_(1)_ = 14.99, *p* = 0.005]: the percentage of mothers holding a university degree was lower in the biHE group.

The biDU and biHE groups differed in the length of uninterrupted HL acquisition (i.e., AoO) as determined by Welch's *t*-test for unequal variance [*t*_(52.21)_ = 5.32, *p* < 0.001]: the children in the biHE had far later SL AoOs. In turn, there were significant differences for the length of exposure to SL, which was calculated as the difference between the chronological age and AoO: a Welch's *t*-test for unequal variance showed [*t*_(61.13)_ = 6.46, *p* < 0.001]. It should be noted that the AoO and LoE were highly correlated in the current sample of children [*r*_(74)_ = 0.90, *p* < 0.001]. There were differences between the two groups concerning the amount of current exposure to HL-Russian [*t*_(69)_ = 4.57, *p* < 0.001]. The bilingual groups differed with respect to family type (HL speaking family vs. mixed family) [χ_(1)_ = 13.07, *p* < 0.001]: in the biDU sample, HL families amounted to 35% of the sample, while in biHE they amounted to 77% of the sample. In the biHE group, 54.3% of the families reported Russian to be the language of communication; while in the biDU group, only 33.3% did; this difference did not reach significance [χ_(1)_ = 3.30, *p* = 0.07].

In the biDU sample, there were no differences in the parental ratings for HL and SL proficiency (i.e., *How would you rate the language proficiency of your child in HL/SL on the scale of 1–4*), as determined by the paired *t*-test [*t*_(36)_ = 0.14, *p* = 0.87], suggesting that the parents viewed their children as balanced bilinguals. Alternatively, in the biHE group, there were significant differences between HL and SL proficiency [*t*_(33)_ = 3.24, *p* = 0.003], thus indicating that the parents reported the HL proficiency of the biHE children as higher compared with the SL proficiency.

To sum it up, the background differences between the two bilingual groups reflect the nature of the Russian-speaking populations in the Netherlands and Israel. These inherent background differences will be included in the analysis to understand which factors are related to the success/failure of case morphology acquisition in bilingual children.

### Tasks

#### The Accusative Case Elicitation Task

**Figure 1:**
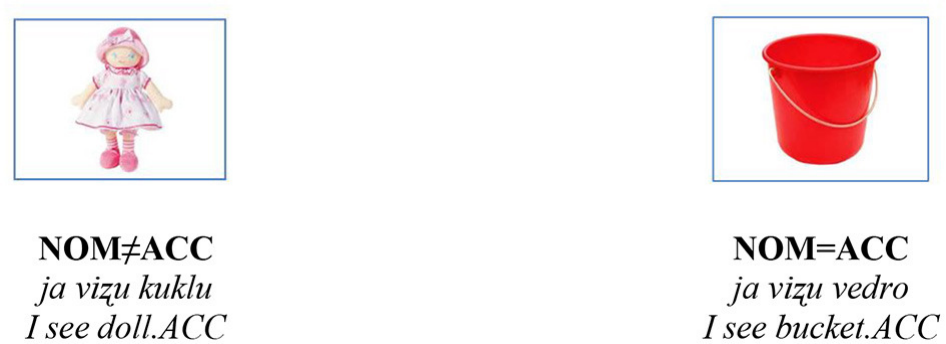
Examples of items used in the ACC and the GEN production tasks.

The ACC case elicitation task investigates the accuracy of ACC inflection production in 36 nouns (Janssen, 2016; Janssen and Meir, 2019). The nouns were all 2- or 3-syllable words, most of the items were taken from the Russian MacArthur-Bates Communicative Development Inventories (Vershinina and Eliseeva, 2007), thus they were all very frequent in child-directed speech, and they were all familiar to young Russian-speaking children. For this task, the pictures were grouped thematically (in groups of four or five items). For example, 3–4 pictures of fruit items (e.g., apple, strawberry, pear) and 3–4 food items (e.g., milk, sausage, egg). The child was asked to describe what he/she sees on the card by saying *ja viʐu ______* “*I* see (target noun).” If the child failed to respond to the sentence with *ja viʐu* ______ “I see ____,” he was reminded to start the sentence with *ja viʐu*. “*I see*.” This was done for each target noun to ensure that the syntactic environment for the ACC case was produced. The task included 12 feminine items, 12 masculine items, and 12 neutral items which varied with respect to whether the ACC form was homophonous or not to the NOM form. See Figure 1.

When the target ACC inflection was produced, the response was coded as “correct” and one point was given. Responses with non-target inflections were coded as “incorrect” and zero points were given. In addition, we noted the type of error in the ACC condition (the use of NOM singular, NOM plural, over-generalization/over-extension of inflections, -u (^*^myl-u), -ov (^*^knig-ov), - i/y (^*^myl-y), -a (arbuz-a).

#### The Genitive Case Elicitation Task

The GEN case elicitation task (Janssen, 2016) examined the production of GEN in the context of negation. The child was asked to name the picture *vot _________* “here is (target noun),” then to turn a picture over and say *a teper net* _________ “and now there is no noun. GEN.” The task included the same 36 items as the ACC case task (see Figure 1). Similar to the ACC task, some nouns in GEN forms are homophonous to the NOM (e.g., *mjaso* “meat”, *jabloko* “apple”).

The responses of the children were coded as “correct” when the target GEN inflection was produced. Responses with non-target inflections were coded as ‘incorrect'. In addition, similar to the ACC task, we noted the type of error in the GEN condition -u (^*^myl-u), -ov (^*^knig-ov), - i/y (^*^myl-y).

### Procedure

Informed parental consent was secured for each child before the testing session, as well as the oral ascent of the child. Each participant was tested individually. The experimenter gave oral instructions. Four warm-up items were administered to familiarize the children with the task: the trial items were not included in the analysis. The study was approved by the Ethics Committee of the University of Amsterdam and the review board of Bar-Ilan University.

The administration of both tasks took an average of around 5 min to complete. The responses of the children were audio-recorded and then transcribed and coded off-line.

## Results

**Figure 2:**
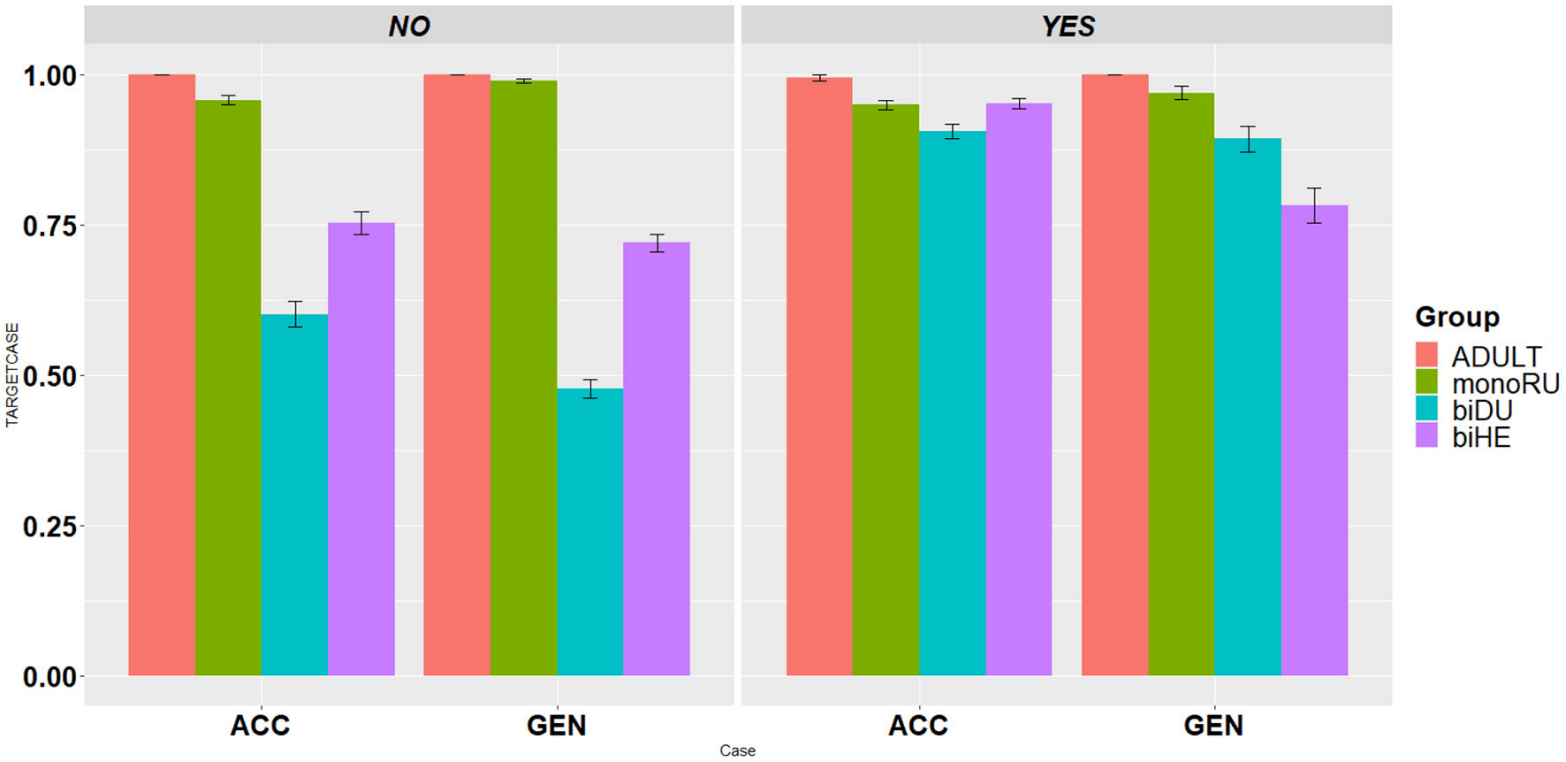
Mean accuracy scores (with standard error bars) per case (ACC vs. NOM) per group (ADULT, monoRU, biDU, biHE) per inflection type (Homophonous to NOM: NO vs. YES).

The descriptive results for the accuracy of the ACC and GEN production are presented in Figure 2 for the child and adult data. The results indicate that monolingual adults show a ceiling performance, confirming our target vs. non-target coding. Similarly, Russian-speaking monolinguals showed a near-ceiling performance (above 0.95) for both ACC and GEN forms. Bilinguals (both biDU and biHE) showed a near-ceiling performance on the forms which are homophonous to NOM in the ACC and GEN contexts, while on the non-homophonous forms which require the use of a dedicated inflection, the performance was lower.

### Effects of Cross-Linguistic Influence in HL Morpho-Syntactic Acquisition

Following previous studies, our first statistical analysis aimed to assess the potential effects of SL properties on HL acquisition by comparing bilinguals and monolinguals.

Given the binary nature of the data (Target/Non-Target), we analyzed the child data using a mixed-effects binomial regression with the group (monoRU, biDU, biHE), case (ACC vs. GEN), inflection type (homophonous to NOM: no vs. yes) as fixed factors. The models were built by adding random and fixed variables in a step-by-step procedure, starting with an intercept-only baseline model. The null models included both by-subject random intercepts and by-stimulus random intercepts. With the inclusion of random slopes, the models failed to converge, and therefore random slopes were not included in the final models. First, we built the model starting with the group, and then we added the case and inflection type as fixed factors. The variables and/or the interactions of the variables were retained in the model only if they significantly improved the fit of the model, resulting in a reduced Akaike information criterion (AIC)-value. We also included a three-way interaction Group^*^Case^*^ Inflection Type. The inclusion of two-way interactions did not significantly improve the fit of the model. The comparison of the models was carried out using one-way ANOVAs, the *p*-value which is higher than 0.05 indicated that the variable and/or the interaction does not improve the goodness of the fit. The analysis was conducted using R (R Core Team, 2020). The final minimal adequate model performed significantly better than the minimal baseline model.

The model summary is presented in Table 3. The results indicated a significant effect on the groups: both bilingual groups were significantly different from their monolingual peers. There was a significant effect on the Case (ACC vs. GEN), and there were significant interactions (Group^*^Inflection Type, Group^*^Case and Group^*^Case^*^Inflection Type).

**Table 3:** Model Summary for target case production for the child data.

	**Estimate**	**SE**	***Z***	**Sig**.
(Intercept)	3.4882	0.317	11.003	<0.0001
Group (monoRU vs. biDU)	−3.1096	0.3828	−8.124	<0.0001
GroupbiHE (monoRU vs. monoRU)	−2.1421	0.3941	−5.435	<0.0001
Case (ACC vs. GEN)	1.6972	0.3372	5.033	<0.0001
Inflection_TYPE (No vs. YES)	0.2603	0.2755	0.945	0.345
Group (biDU) *Inflection TYPE (YES)	2.4342	0.3119	7.804	<0.0001
Group (biHE)* Inflection TYPE (YES)	2.5873	0.3481	7.433	<0.0001
Group (biDU)*Case (GEN)	−2.2181	0.3567	−6.219	<0.0001
Group (biHE)*Case (GEN)	−1.6742	0.3636	−4.605	<0.0001
Case (GEN)*Inflection TYPE (YES)	−1.5356	0.5422	−2.832	<0.001
Group (biDU)*Case (GEN)*Inflection TYPE (YES)	1.562	0.6022	2.594	<0.001
Group (biHE)*Case (GEN)*Inflection TYPE (YES)	−1.0611	0.6114	−1.736	0.082
Observations	7,513			
Marginal R2/Conditional R2	0.379/0.618			

As a follow-up on the significant three-way interaction (see Table 4), pair-wise comparisons with an adjusted alpha level using the Tukey method showed that on the non-homophonous forms, i.e., the forms that require the use of a dedicated infection to mark ACC/GEN cases, both bilingual groups were significantly less accurate as compared with their monolingual peers. As for the forms, which are homophonous to NOM, the biDU did not differ from monolingual controls, while the biHE differed only on the GEN forms. Thus, quantitative differences emerged between monolinguals and the two bilingual groups on nouns that require dedicated inflections in ACC and GEN forms which are different from NOM ones.

**Table 4:** Pairwise contrasts for group comparisons (monoRU vs. biDU, monoRU vs. biHE) per case per inflection type.

**Case**	**Inflection type (homophonous to NOM)**	**Group pairwise contrasts**	**Contrast estimate**	**SE**	***Z***	**Adj. Sig**.
GEN	NO	**monoRU—biDU**	5.328	0.419	12.724	<0.0001
		**monoRU—biHE**	3.816	0.427	8.928	<0.0001
	YES	monoRU**—**biDU	1.332	0.536	2.485	0.350
		monoRU**—**biHE	2.290	0.527	4.347	0.0008
ACC	NO	**monoRU—biDU**	3.110	0.383	8.124	<0.0001
		**monoRU—biHE**	2.142	0.394	5.435	<0.0001
	YES	monoRU**—**biDU	0.675	0.381	1.773	0.833
		monoRU**—**biHE	−0.445	0.415	−1.073	0.996

To shed further light on the contribution of SL properties to HL acquisition, we compared the accuracy of production of ACC and GEN cases in the three child groups (monoRU, biDU, and biHE). The adjusted pair-wise comparisons using the Tukey method indicated that while monolingual controls were more accurate on the GEN forms, the two bilingual groups were more accurate on the ACC forms as compared with the GEN ones (see Table 5). It is worth noticing that the gap between ACC and GEN was more likely to be observed in the biHE group.

**Table 5:** Pair-wise ACC-vs-GEN contrasts for monoRU, biDU and biHE groups.

**Group**	**Contrast estimate**	**SE**	***Z***	**Adj. Sig**.
monoRU	−0.929	0.269	−3.455	0.0073
biDU	0.508	0.163	3.110	0.0230
biHE	1.275	0.173	7.386	<0.0001

**Figure 3:**
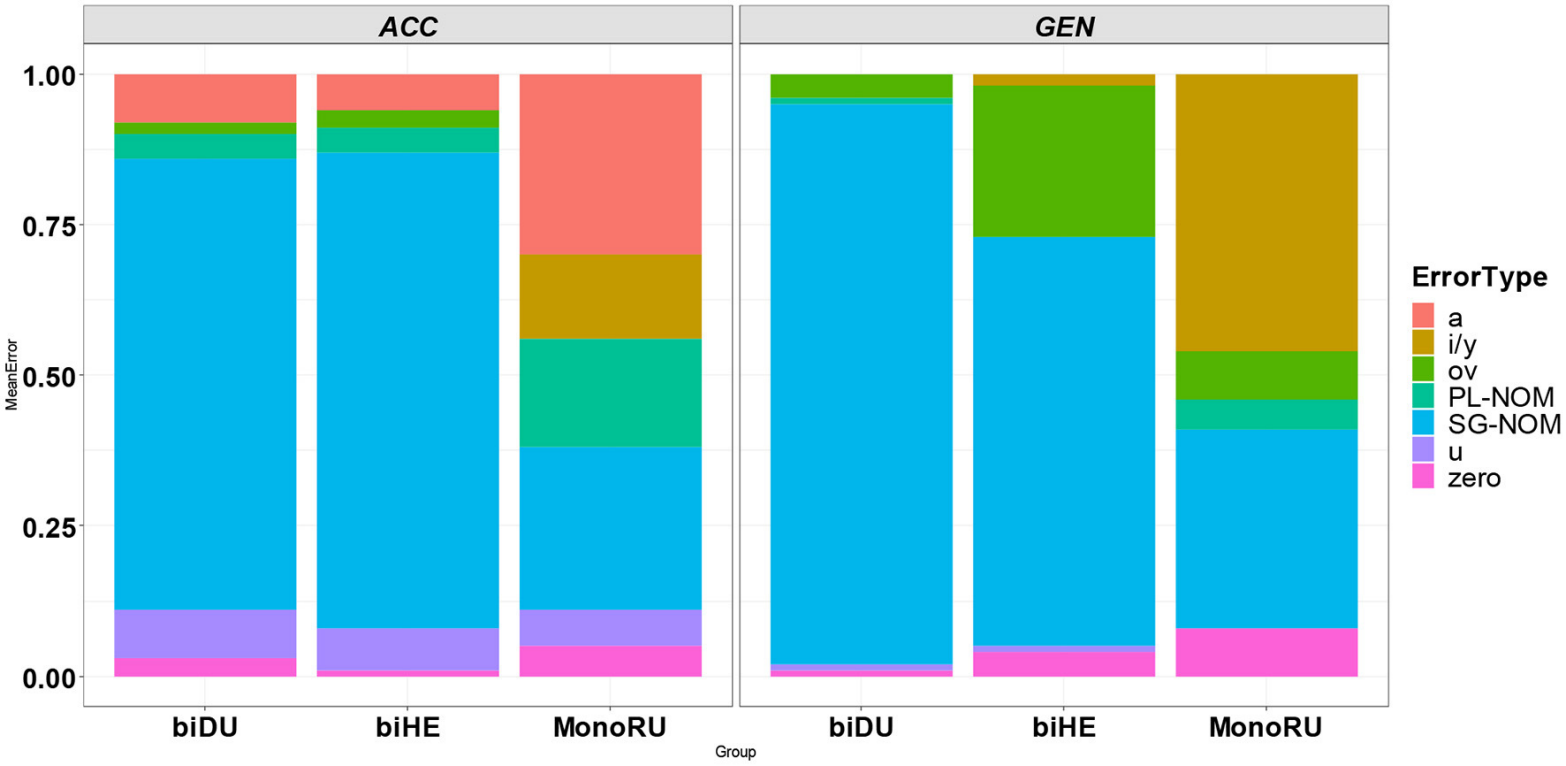
Error pattern distributions per case per group.

Subsequently, we conducted a qualitative analysis of error patterns across the three child groups to shed light on whether monolingual-bilingual group differences were only quantitative or also qualitative (see Figure 3), i.e., whether bilingual and monolingual children resorted to different types of errors. Indeed, the error pattern analysis revealed that there are qualitative differences: the most common error pattern among bilingual children was the use of default NOM forms in ACC and GEN contexts (biDU: ACC = 0.75; GEN = 0.92; biHE: ACC = 0.79; GEN = 0.68). In the monolinguals, errors were very infrequent totaling 87 (63 in the ACC contexts and 24 in the GEN contexts): the use of NOM forms was not the most prevalent type of error (ACC = 0.27; GEN = 0.33). The erroneous substitution of other non-target inflections was more common in the monoRU group leading to innovations (e.g., ACC: *a teper' net* ^*^promidory / ^*^myly / ^*^mjasy; GEN: ja vižu ^*^jabloku / ^*^jajcu).

In contrast, bilingual children resorted to NOM forms in both ACC contexts (*ja vižu* ^*^kukla / ^*^klubnika/ ^*^krokodil / ^*^butylka ‘I see doll.NOM” / strawberry.NOM / crocodile.NOM / bottle.NOM), as well as in GEN contexts (*a teper' net* ^*^gruša / ^*^karandas/ ^*^ *kolbasa ‘*and there is no pear.NOM /pencil.NOM/ sausage.NOM').

To sum up, the results of the first analysis, which compared monolinguals vs. bilinguals, showed that bilingual children were less accurate than their monolingual peers on nouns that require the use of dedicated ACC and GEN inflections (i.e., non-homophonous inflections). Furthermore, unlike monolingual child controls who were more accurate on the GEN forms as compared with the ACC forms, the picture was reversed in the bilingual groups. We predicted the gap to be observed only in the biHE group, due to the inherent properties of SL-Hebrew which mark the ACC case, but not in the biDU group. It is important to note that the gap between ACC and GEN was more likely to be detected in the biHE group. In addition, both bilingual groups showed different error profiles as compared with monolinguals. While monolinguals substituted the target forms with other non-target inflected forms, bilinguals favored the use of NOM forms.

In our subsequent analyses, we focused on the two bilingual groups. First, we evaluated the contribution of language-external variables in bilingual HL development. Second, we attempted to assess the contribution of cross-linguistic influence and language-external factors.

### The Effects of Language-External Factors

**Figure 4:**
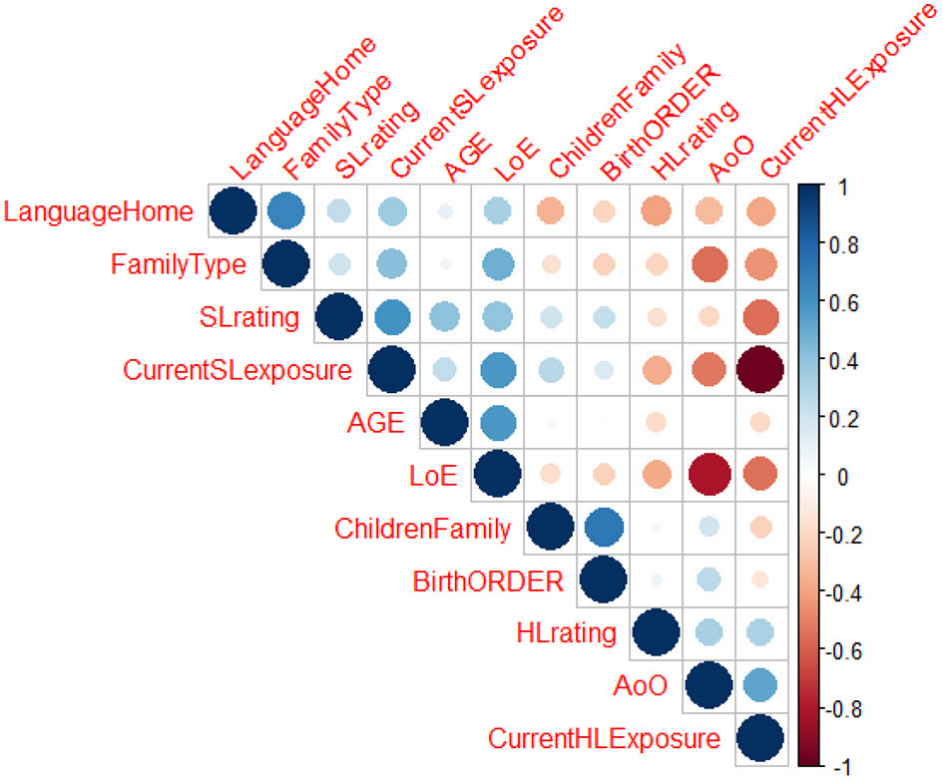
Correlations between language-external factors. Positive correlations are displayed in blue and negative correlations in red. The color intensity and the size of the circle are proportional to the correlation coefficients. Insignificant correlations are removed from the correlational matrix (*p* < 0.01).

To address our second research question on the role of language-external factors in bilingual HL development, we first looked at the interrelationship between various language-external factors. This was done to determine which factors should be included in the subsequent statistical models. The results indicated medium to strong correlations between different language-external factors (see Figure 4): age, AoO, length of exposure to SL (LoE), language spoken at home, family type, current exposure to HL and SL, parental rating of child HL and SL proficiency, number of children in the family, and first-born or not. For example, the results indicated that there were strong negative correlations between the current HL and SL exposure indices (*r* = −0.98), suggesting that children with increased current exposure to HL have less exposure to SL. There were strong negative correlations between AoO and LoE (*r* = −0.81): children with earlier AoO had more exposure to L2. Thus, following the observed correlations between some of the language-external factors, it was decided to exclude some variables from further analyses (if the correlation between the two variables was above 0.7).

To address the more specific aspect of our second research question concerning the role of language-external factors in HL morphosyntactic acquisition, as in the previous model we started with case production accuracy as the outcome variable coded in a binary manner (Target/Non-Target), and random factors (Participant and Item). We included case and inflection type, as well as language-external variables. The best model included case and inflection type, plus AoO and HL parental rating and the interactions between inflection type and AoO, and inflection type and HL parental rating (see Table 6). We note that chronological age, type of family, current exposure to HL, number of children in the family, and the status of being firstborn did not improve the fit of the model, therefore these variables were excluded from the final model.

**Table 6:** Model summary for target case production and language-external factors.

	**Estimate**	**SE**	***Z***	**Sig**.
(Intercept)	−2.655	0.681	−3.896	<0.0001
Inflection Type (yes/no)	6.146	0.487	12.615	<0.0001
Case (GEN vs. ACC)	−0.551	0.104	−5.274	<0.0001
AoO	0.029	0.010	2.814	0.006
HL rating	1.031	0.230	4.482	<0.0001
Inflection Type (yes/no) * AoO	−0.013	0.007	−1.964	0.049
Inflection Type (yes/no) * HL rating	−1.194	0.154	−7.757	<0.0001
Observations	4,383			
Marginal R2/Conditional R2	0.304/0.566			

**Figure 5:**
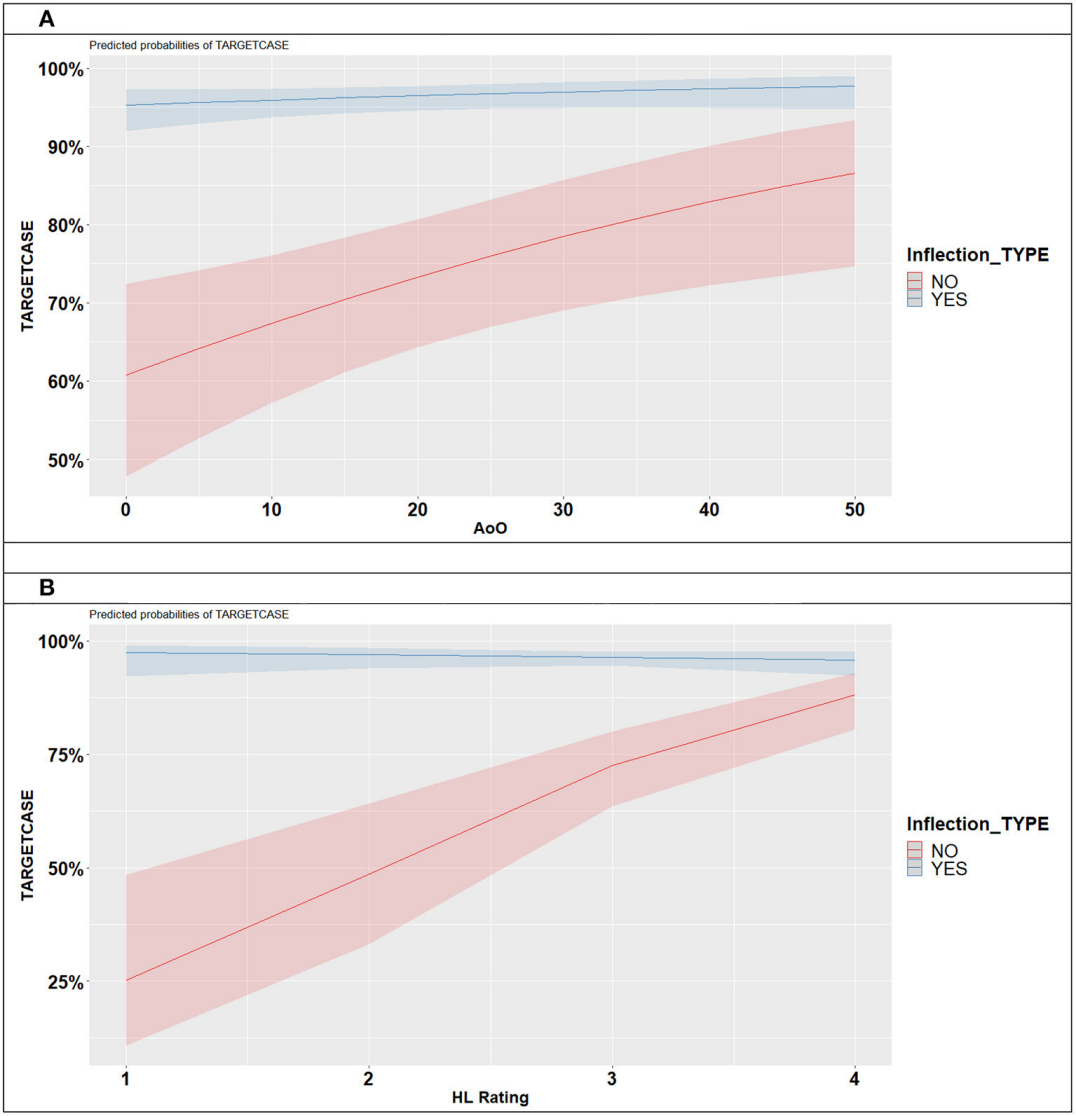
**(A)** Predicted case production by inflection type by AoO. **(B)** Predicted case production by inflection type by HL rating.

The results of the model are illustrated in Figures 5A,B. Language-external factors affect the case accuracy production of nouns which require the use of dedicated case inflections only. While language-external factors do not affect forms that are homophonous to NOM, the accuracy of the case production (which requires the use of a dedicated inflection, ACC and/or GEN) is predicted by AoO and HL ratings. Children with later AoOs of bilingualism are more likely to produce target case inflections (see Figure 5A). The same applies to HL rating: with the growing proficiency in HL, the accuracy of the target case inflections improves (see Figure 5B). To summarize, the production of target case inflections increases with later AoOs of bilingualism and growing proficiency in HL.

### The Effects of Cross-Linguistic Influence and Language-External Factors

Finally, to address our third research question which aimed to evaluate the interplay between cross-linguistic influence and language-external factors in bilingual children, we further compared the two bilingual groups. These two groups (biDU and biHE) varied with respect to the case properties in the SL. While the Dutch language does not mark cases morphologically on nouns, the Hebrew language has a dedicated particle “*et*” to mark the ACC case. Thus, the purpose of our third model was to evaluate the contributions of SL factors and language-external factors to HL morphosyntactic acquisition. Based on the two previous models, we fitted a model with case production accuracy as the outcome variable, and participant and item as random factors. We attempted to assess the presence of three-way interactions between case (ACC vs. GEN), AoO, and group (biDU vs. biHE), on one hand, and between case (ACC vs. GEN), HL Rating, and group (biDU vs. biHE), on the other hand. While the three-way Group^*^Case^*^AoO interaction did not improve the fit of the model, the Group^*^Case^*^HL rating did significantly improve it. The estimates of the final model are presented in Table 7.

**Table 7:** Model summary for target case production (cross-linguistic influence and language-external variables).

	**Estimate**	**SE**	***Z***	**Sig**.
(Intercept)	−1.384	0.835	−1.657	0.098
Inflection Type (yes vs. no)	2.452	0.170	14.483	<0.0001
Case (GEN vs. ACC)	−0.413	0.421	−0.982	0.326
Group	−0.667	1.594	−0.418	0.676
HL rating	0.668	0.289	2.312	0.021
Case (GEN) * Group (biHE)	−1.970	0.848	−2.322	0.020
Group (biHE) * HL rating	0.378	0.489	0.774	0.439
Case (GEN) * HL rating	−0.076	0.151	−0.500	0.617
Case (GEN) * Group*HL rating	0.686	0.267	2.575	0.010
Observations	4,383			
Marginal R2/Conditional R2	0.330/0.577			

**Figure 6:**
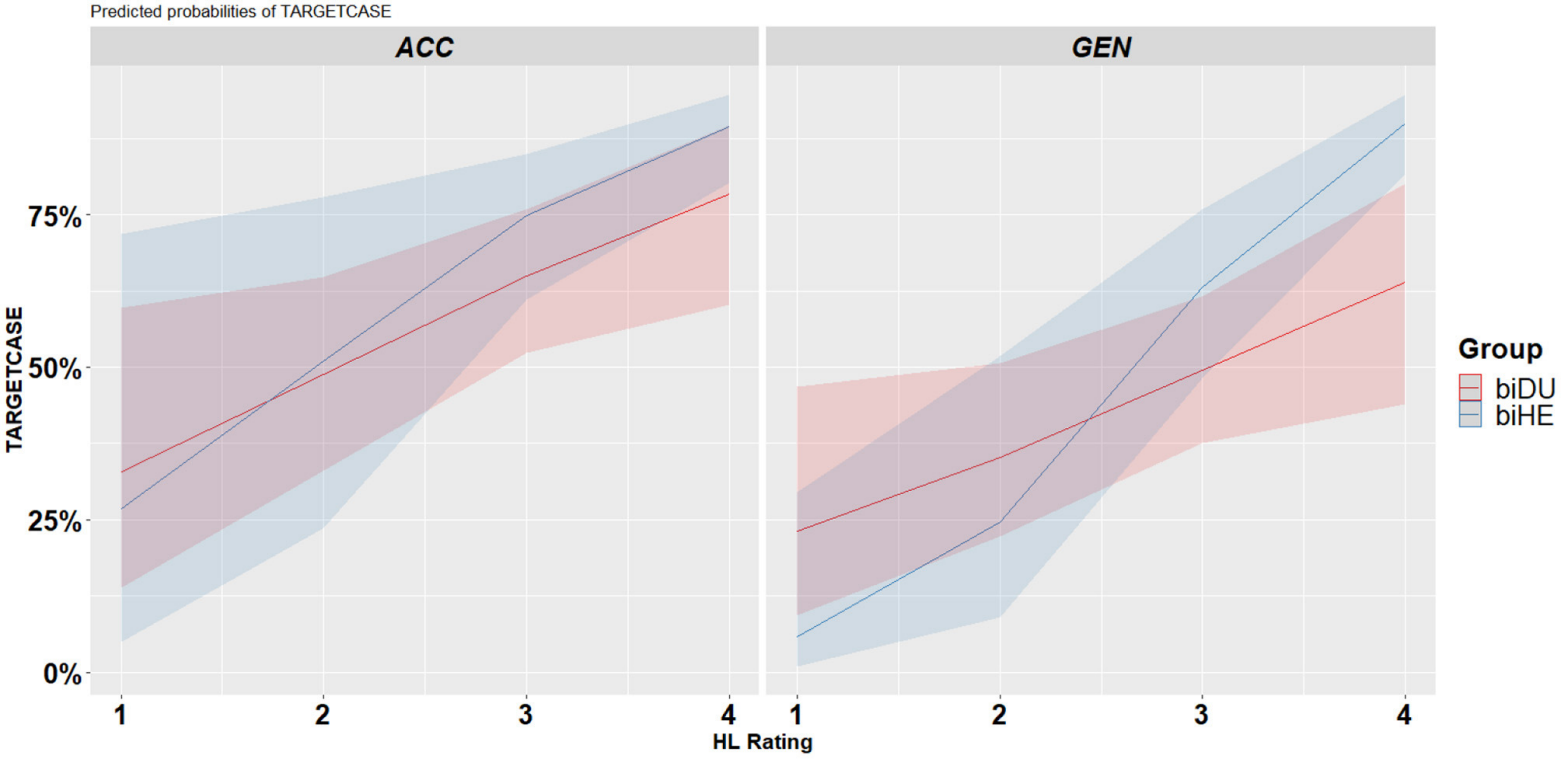
Predicted case production by case by group by HL proficiency.

The three-way interaction indicated that there is an interplay between language-external factors and cross-linguistic influence. The growth in the biHE group is steeper for the ACC case as compared with the biDU group which might indicate the positive direct effect of ACC marking in the Hebrew language. Furthermore, there is also a steep growth for the GEN case in the biHE group, which might indicate an indirect positive effect from the SL that marks cases morphologically. In the biDU, the increase in HL proficiency is associated with the parallel growth for ACC and GEN, with no advantage for ACC (see Figure 6).

## Discussion

The current study aimed to investigate the potential mechanisms affecting child HL development. HL speakers are exposed to their HL from birth *via* naturalistic input. Adult HL speakers were reported to show divergences in a number of language domains with morphosyntax being the hallmark of difficulties in HL adult grammars (for an overview see Montrul, 2016; Polinsky, 2018). The current study employed monolingual vs. bilingual comparisons, as well as between-bilingual-group comparisons to contribute to the knowledge on the development of child HL grammar. Most of the previous studies investigating cross-linguistic influence compared the performance of bilinguals with that of monolinguals; fewer carried out multiple bilingual group comparisons. Van Dijk's (2021) meta-analysis investigating cross-linguistic influence via bilingual vs. monolingual comparisons confirmed the presence of cross-linguistic influence in the acquisition of morphosyntax based on 26 studies. However, some studies employing between-bilingual-group comparisons did not find SL influence on HL and point to the effect of input characteristics (see the introductory section of this paper). In line with previous studies, our results revealed a complex picture of HL development.

Firstly, the results of the current study showed that the domain of morphosyntax is not only challenging for adult HL-Russian speakers (see Polinsky, 2018 and references in it), but also for child HL-Russian speakers. Monolingual Russian-speaking children showed a near-ceiling performance in line with the previous literature (e.g., Gvozdev, 1961; Babyonyshev, 1993; Protassova and Voeikova, 2007; Cejtlin, 2009). In contrast, both bilingual groups were less accurate in case morphology compared to their monolingual peers. This has been shown for the ACC case as well as the GEN case. We also found different error patterns in the bilingual groups. While monolingual Russian-speaking controls produced non-target inflected forms, bilingual HL-Russian speakers resorted to the default NOM forms. The overuse of NOM was the most common error in the bilingual groups. The results of the current study corroborate previous findings for child HL-Russian speakers (see Turian and Altenberg, 1991; Gagarina, 2011; Ringblom, 2012; Schwartz and Minkov, 2014; Janssen, 2016; Meir et al., 2017; Protassova et al., 2017; Gagarina and Klassert, 2018; Janssen and Meir, 2019; Armon-Lotem et al., 2021). Most of the previous studies concluded that the reduced accuracy in case morphology is driven by the properties of the SLs, as the SLs do not mark case morphology in nouns. This is indeed a plausible explanation, and our study does not rule out this option.

To deepen our understanding of the mechanisms of HL development, we conducted a comparison between the two bilingual groups, beyond the monolingual vs. bilingual comparisons. Studies comparing different bilingual groups matched on one language are scarce. The two bilingual groups in this study differed with respect to the SL properties. Dutch does not mark cases morphologically on nouns. Hebrew marks ACC case with the particle “*et*” in font of definite nouns. Thus, we hypothesized that if the presence/absence of a feature in the language affects HL acquisition, Russian-Hebrew bilinguals would show better mastery of the ACC case as compared with the GEN case in HL-Russian. No ACC-GEN asymmetry was expected in the bilingual Russian-Dutch group if the properties of SL shape HL acquisition. Alternatively, under the *feature (re)-assembly hypothesis* (Lardiere, 2009), we predicted no facilitative effect for the acquisition of ACC in Russian-Hebrew bilinguals, since the ACC case is mapped onto different lexical categories in the two languages of Russian-Hebrew bilinguals (onto case inflection in Russian and particle *et* in Hebrew). Under this scenario, no asymmetry was expected between the ACC and GEN case production. Similar to previous studies, multiple bilingual group comparisons yielded complex results. First, the comparison of the case production in HL-Russian among Russian-Dutch and Russian-Hebrew bilinguals showed an advantage for the ACC forms in both groups, while in the monolingual controls, the reverse picture was observed, with better performance on GEN forms. Based on the frequency of ACC and GEN forms in the general corpus, no differences were expected. However, our study did not include the frequencies of ACC and GEN form in the child-directed speech. Future studies need to include frequencies of forms in child-directed speech to further shed light on the mechanisms of HL development, thus focusing more on quantitative and qualitative input characteristics.

Language-external variables play an important role in monolingual language acquisition and even more so in bilinguals (see Armon-Lotem and Meir, 2019). Thus, it is plausible to suggest that monolingual vs. bilingual differences (also observed in the current study) are not solely driven by the properties of the SL, but also related to language-external variables. Indeed, recent studies comparing multiple bilingual groups showed that it is not cross-linguistic influence that shapes HL development, but rather, for the most part, language-external variables (see Rodina et al., 2020). The current study confirms that language-external variables are an important part of the puzzle of bilingual HL development. The findings show that the AoO of bilingualism and HL proficiency (as indexed by HL parental ratings) affect the HL morphosyntactic development. Longer periods of uninterrupted acquisition of HL and growing HL proficiency contribute to the success of the HL morphosyntactic development, and as a result language-external factors compensate for the possible negative effects of the SL on the HL.

Interestingly, it was AoO, rather than chronological age, that predicted case production in the HL. The effect of chronological age is robust in the monolingual acquisition, yet not so strong in bilingual HL and SL morphosyntactic development. Our findings showed that chronological age had no effect on HL morphosyntactic development, in line with the results of the meta-analysis by Van Dijk et al. (2021) which showed that age does not module the extent of cross-linguistic influence. However, AoO has been shown to be one of the key factors shaping HL development in bilinguals in previous studies investigating SL and HL morphosyntactic development (see Tsimpli, 2014; Meir et al., 2017; Armon-Lotem et al., 2021). In addition, we found that HL proficiency is a powerful predictor of case production in HL. As the HL proficiency increases, children are more likely to have a target-like case system. This has also been related to HL and SL input/exposure factors. As previously noted, as HL speakers grow older, they switch from their HL to the SL, which is visible in the increased SL input/exposure and growing SL proficiency, while there is a simultaneous decrease in HL input exposure. Indeed, our correlational analyses showed that HL proficiency was related AoO, current HL exposure, and family type. All these variables contribute to a higher level of HL proficiency (see Figure 4).

Furthermore, our study attempted to evaluate the interplay between cross-linguistic variables and language-external factors, i.e., trying to evaluate the mitigators of cross-linguistic influence. The meta-analysis by Van Dijk et al. (2021) investigating language dominance as a possible moderator of cross-linguistic influence, showed that that the effect of the SL (the dominant language) on the HL (the non-dominant language) is stronger compared with the influence in the opposite direction. The authors showed that the other indices of dominance (e.g., amount of language exposure and use, lexical proficiency, and fluency ratings by parents or teachers) did not detect the interaction between these dominance measures and cross-linguistic influence. In our study, the interaction between cross-linguistic influence and HL proficiency as indexed by parental ratings was observed. Our study showed that the growth is steeper across different proficiency levels when there is some overlap between the two languages. It should be noted that HL proficiency was closely related to the language spoken at home, AoO, and HL current input.

Previous attempts to model HL development, i.e., to distill the mechanisms influencing ultimate HL attainment, have arrived at different conclusions. Some scholars suggest that insufficient input is the key mechanism driving changes in HL grammar. Others believe that cross-linguistic influence is the sole mechanism shaping the ultimate HL attainment. The results of our study are in line with the proposals advocating for an integrative approach in modeling HL development. Cross-linguistic influences on HL are modulated by language-external factors (see Daskalaki et al., 2020; Van Dijk et al., 2021). Our findings show that being exposed to HL from birth does not guarantee the setting of parameters in line with monolingual grammar. The (re)-setting of parameters in HL is influenced by the presence/absence and realization of the parameters in the SL [as predicted by the *feature (re)-assembly hypothesis* (Lardiere, 2009)]. But we also showed that there are individual differences in the (re)-setting of the parameters, which are related to language-external factors. Putnam and Sánchez (2013) previously proposed that the (re)-setting of the feature parameters in HL is related to the feature activation in production and comprehension. The authors propose that HL speakers go through various stages in HL acquisition depending on the frequency of the feature activation. At Stage 1, HL speakers (re)-assemble some of their HL features in production under the influence and activation of SL features. At Stage 2, more HL features undergo re-assembly, including lexical items. At Stage 3, HL speakers show difficulties with HL features in production. Finally, at Stage 4, HL feature activation is impeded not only in production but also in comprehension. Our findings indirectly support this claim suggesting that the HL feature activation in production is very closely related to the characteristics of the language-external factors. HL speakers with more input/exposure have more opportunities to activate their HL features, as compared with those who have less input/exposure to HL and as a result, more input/exposure to SL. The decreased HL exposure and decreased HL proficiency affect the frequency of the activation of HL, which results in the re-assembly of features in some speakers under diminished HL input and subsequent lower language proficiency.

## Conclusions, Applied Implications, and Future Research

The results of the current study investigating morphosyntactic development in HL paint a complex picture. Our results show that HL acquisition is impeded under the influence of the properties of SL which is evident in the lower performance of both bilingual groups as compared with the monolingual controls, who showed a near-ceiling performance on the production of target inflections in ACC and GEN contexts. More specifically, the acquisition of morphology is impeded when there are differences in the mapping of the functional features (as in the case with Russian-Hebrew bilingualism) and/or the absence of this feature marking (as in the case of Russian-Dutch bilinguals). The findings showed language-external factors as important factors modulating the potential negative effects of cross-linguistic influence. More specifically, the results show that AoO and HL proficiency play an important role in mitigating the possible effects of cross-linguistic influence. Finally, the study shows an interaction between SL properties and language-external factors: grammatical features which have more resemblance in the HL and the SL might be acquired faster under direct or indirect transfer from the SL. Thus, HL development is an intricate interplay between cross-linguistic influence and language-external factors.

The findings of the current study have important clinical and pedagogical implications. Firstly, on the clinical side, the study shows that the HL of bilingual children exhibits quantitative and qualitative divergences from the monolingual controls. Thus, clinicians need to be cautious when assessing and interpreting the assessment results of bilingual children in their HL (see Armon-Lotem et al., 2021). Secondly, the study shows a complex picture of HL development emphasizing the importance of HL input/exposure. Previous research showed that the parents of child HL speakers are generally interested in transmitting their HL to their children [see (Otwinowska et al., 2021)]—they see it as an integral part of maintaining a positive relationship with parents, grandparents, and extended families. Thus, efforts should be made to maintain HL input/exposure. Our findings confirmed that the HL “will not take care of itself” (Mieszkowska et al., 2017), as the HL is in danger of turning into a weaker language.

While our study makes a substantial contribution to the understanding of child HL development, it is not without limitations. First, our study focused only on the production of ACC and GEN cases, therefore, future studies should combine both production and comprehension tasks to deepen our understanding of the mechanisms of HL morphosyntactic acquisition. The investigation of production and comprehension of the same phenomenon in HL speakers would enable future studies to identify the developmental stage of the bilingual speaker. This would shed light on whether the problems are present only at the level of production, or if comprehension is also affected. Future research needs to focus more on the individual differences which shape HL development, including various linguistic, extra-linguistic, and cognitive measures. The inclusion of cognitive measures might help us understand how domain-general mechanisms (such as working memory and inhibition) modulate cross-linguistic competition. Finally, the current study was based on the participation of children with sparse case morphology in their SL. Future research should be extended to the bilingual-group comparisons of children speaking SLs that have rich case morphologies which are realized with case inflections (e.g., Finnish, Latvian, Estonian, Ukrainian, Hungarian, Turkish), and SLs that mark case morphology differently from Russian (e.g., German, Greek). Studies comparing multiple bilingual groups will further deepen our understanding of the interplay between cross-linguistic influence and language-external factors.

## Data Availability Statement

The raw data supporting the conclusions of this article will be made available by the authors, without undue reservation.

## Ethics Statement

The studies involving human participants were reviewed and approved by Bar-Ilan University, The University of Amsterdam. Written informed consent to participate in this study was provided by the participants' legal guardian/next of kin.

## Author Contributions

BJ carried out the collection of the data with monolingual participants and bilingual Russian-Dutch bilinguals. NM carried out data collection with bilingual Russian-Hebrew bilinguals and was responsible for data analysis and interpretation of the results. Both authors shared responsibility for drafting of the work, final approval of the version to be published, and the conception of the work.

## Conflict of Interest

The authors declare that the research was conducted in the absence of any commercial or financial relationships that could be construed as a potential conflict of interest.

## Publisher's Note

All claims expressed in this article are solely those of the authors and do not necessarily represent those of their affiliated organizations, or those of the publisher, the editors and the reviewers. Any product that may be evaluated in this article, or claim that may be made by its manufacturer, is not guaranteed or endorsed by the publisher.
